# Multifidus degeneration correlates with the extent of L4 translation in degenerative lumbar spondylolisthesis

**DOI:** 10.1016/j.bas.2026.106272

**Published:** 2026-08-07

**Authors:** Maximilian Muellner, Lukas Schönnagel, Ulrike Kielburg, Hendrik Schmidt, Alexander P. Hughes, Matthias Pumberger, Friederike Schömig

**Affiliations:** a Center for Musculoskeletal Surgery, Charité – Universitätsmedizin Berlin, Charitéplatz 1, Berlin, 10117, Germany; b Julius Wolff Institute, Berlin Insitute of Health, Charité – Universiätsmedizin Berlin, Augustenburger Platz 1, Berlin, 13353, Germany; c Department of Orthopaedic Surgery, Hospital for Special Surgery, Weill Cornell Medicine, New York City, NY, USA

**Keywords:** Degenerative lumbar spondylolisthesis, Paraspinal muscles, Spine surgery, Degeneration

## Abstract

**Introduction:**

While the role of paravertebral muscles has increasingly been studied in the pathogenesis and outcome of spinal pathologies, their role in the development of degenerative lumbar spondylolisthesis (DLS) remains poorly understood. Our study's aim was to analyze whether the extent of L4 translation is associated with changes in paraspinal muscle morphology.

**Material and methods:**

We retrospectively included patients treated surgically for degenerative lumbar spondylolisthesis at the L4/5 level, all of whom underwent single-level surgical intervention solely at this segment. Muscle measurements were performed in magnetic resonance imaging (MRI) and included the cross-sectional area (CSA), functional CSA (fCSA), and fatty infiltration (FI) of the multifidus, erector spinae, and psoas muscles. Radiographs were analyzed for the degree of spondylolisthesis according to Meyerding and the extent of L4 translation in mm. Pearson correlation coefficient was determined to assess the relationship between muscle parameters and L4 translation. A multiple linear regression model was performed, with L4 translation as the dependent variable.

**Results:**

Ninety-six patients were included, 61 (63.5%) were female. Female patients showed a significantly higher frequency of spondylolisthesis Meyerding grades > 1 (p = 0.018). In the psoas, female patients showed a significantly lower CSA (9.61 ± 3.18 vs. 12.32 ± 3.47, p < 0.001) and fCSA (9.50 ± 3.12 vs. 12.12 ± 3.44, p < 0.001). The multiple linear regression model showed a significant positive association between the multifidus FI and L4 translation (b = 0.084, p = 0.009).

**Conclusions:**

We demonstrate an association between multifidus FI with the degree of L4 translation in DLS. Muscle morphology therefore needs to be taken into consideration in both the conservative and the surgical treatment which may be supported by specific multifidus strengthening.

## Highlights

• A retrospective cohort of 96 patients undergoing L4/5 fusion between 2010 and 2022 was analyzed.
• MRI-based measurements included cross-sectional area, functional cross-sectional area, and fatty infiltration of the multifidus, erector spinae, and psoas muscles.
• Multifidus fatty infiltration was significantly associated with L4 translation (b = 0.084, p = 0.009).
• Female patients showed higher rates of Meyerding grade > 1 and significantly lower psoas CSA and fCSA (p < 0.001).
• Multifidus degeneration may contribute to spinal instability and support targeted multifidus strengthening in conservative and surgical planning.

## 1 Introduction

Degenerative lumbar spondylolisthesis (DLS) is among the most common indications for lumbar spine surgery and is defined as an acquired vertebral subluxation, most frequently as an anterior displacement of L4 on L5, with an intact posterior arch (Pumberger et al., 2012; Wiltse, 1981). Due to the degeneration of the intervertebral disc, the facet joints are increasingly loaded which results in segmental hypermobility, facet joint arthropathy, and ultimately in spinal canal stenosis, spondylolisthesis, and progressive deformity (Lee and Patel, 2013; Rangwalla et al., 2023). Clinically, patients present with low back pain, typically in combination with leg pain or radiculopathy and claudication.

While in symptomatic patients with failed conservative treatment the benefits of surgical treatment have been well-established in treating DLS, there still is controversy regarding the optimal surgical strategy with the roles of decompression alone versus decompression with segmental stabilization remaining debated (Schönnagel et al., 2023). Furthermore, there still is a considerable number of patients with persistent disability even after surgery (Abdu et al., 2018; Weinstein et al., 2009; Ghogawala et al., 2016; Austevoll et al., 2020).

Recently, the role of the paravertebral muscles, compromised of the posterior multifidus and erector spinae as well as the anterior psoas muscle, has increasingly been studied in the pathogenesis and treatment of spinal pathologies. Their association with bone quality (Haffer et al., 2023; Muellner et al., 2023), spinal alignment (Muellner et al., 2022), and postoperative complications including the development of proximal junctional failure, screw loosening, non-union, and adjacent segment disease (Pinter et al., 2023; Han et al., 2023) has also been shown in previous analyses.

To determine muscle health, both the cross-sectional area (CSA) and the fat area (FAT) are measured by magnetic resonance imaging (MRI), and muscle degeneration is characterized by a reduced CSA and increased inter- and intra-muscular fat which results in a higher percentage of fatty infiltration (FI) (Kalichman et al., 2017). The FI is considered as an indicator for muscular function (Rahemi et al., 2015; Addison et al., 2014).

As there are increasing data showing that muscular changes also correlate with individual patient outcomes, the evaluation of paraspinal muscle morphology may help in identifying patients who will benefit less from surgical intervention (Han et al., 2023). In this regard, identifying risk factors associated with reduced muscle health preoperatively may aid in developing more individualized treatment plans for affected patients.

The aim of this study aim therefore was to analyze whether the extent of L4 translation in DLS is associated with changes in paraspinal muscle morphology.

## 2 Material and methods

### 2.1 Patient inclusion

This retrospective study was approved by the hospital's institutional review board (EA2/194/22). Individual informed consent was waived due to the retrospective nature of the study. The investigation was in accordance with the ethical principles of the Helsinki Declaration and the Strengthening the Reporting of Observational Studies in Epidemiology (STROBE) guidelines were followed in the reporting of this study.

A retrospective review was performed using the hospital's medical records system to identify patients undergoing posterior fusion for spondylolisthesis of the L4/5 level between January 2010 and December 2022. Exclusion criteria were patient age < 18 years, any previous lumbar spine surgery, fusion on levels other than L4/5, and missing or non-measurable MRI scans and/or radiographs. Patient demographics including age, sex, height, weight, body mass index (BMI), comorbidities and American Society of Anesthesiology (ASA) score were retrieved from the electronic patient records.

### 2.2 Muscle measurements

**Fig. 1:**
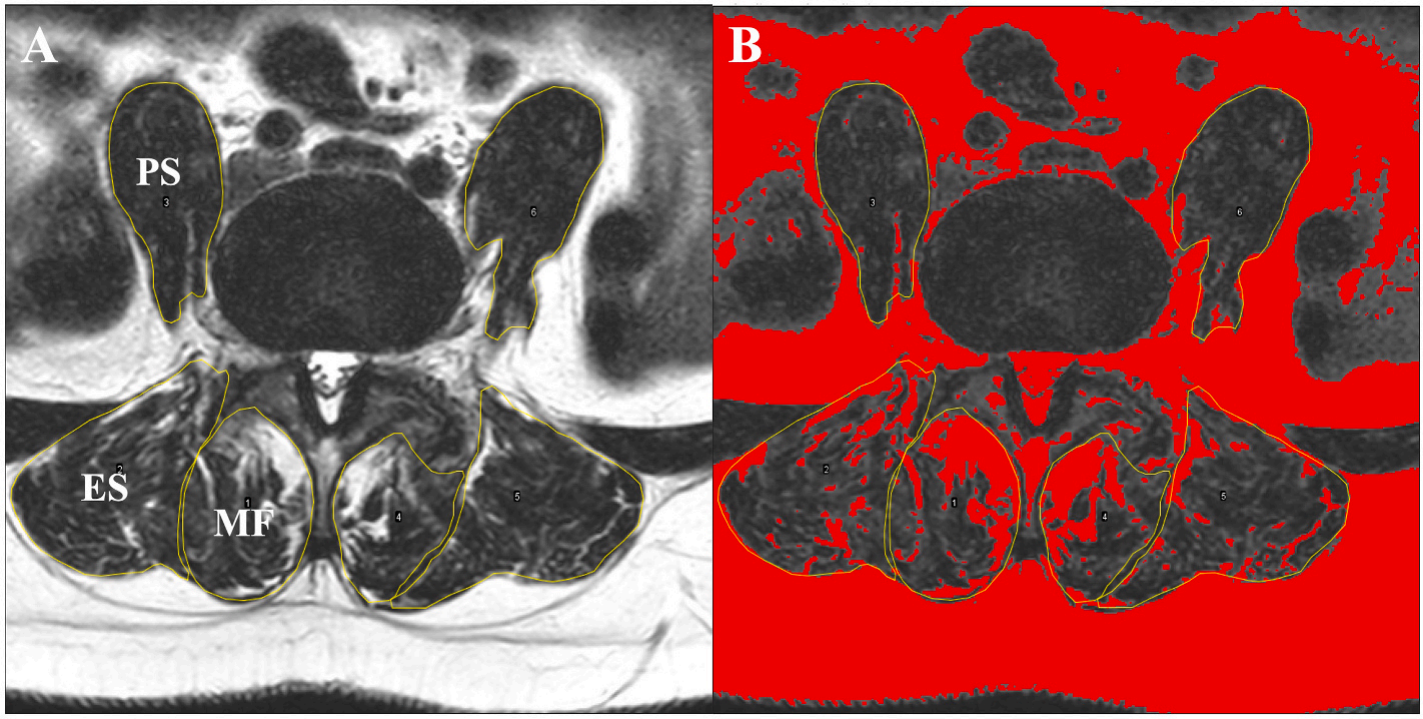
Illustration of the muscle measurement technique. Image A demonstrates the segmented muscles (ES = M. erector spinae; MF = *M. multifidus*; PS = M. psoas). Image B shows the threshold values for the pixel intensity. The red area in the previously selected regions of interests is interpreted as fat.

The muscle measurements were performed at mid-disc level of L4/5 by utilizing T2-weighted axial images (Fig. 1). Regions of interest (ROI) were the left and right psoas muscle (PS) as well as the posterior paraspinal muscles including the multifidus (MU) and erector spinae (ES) muscles.

In the defined ROI, a free software program was used (ImageJ, version 1.53o, National Institutes of Health, Bethesda, Maryland, USA) to determine the CSA, cm^2^, the functional CSA (fCSA, cm^2^), which is considered as lean muscle, and the intramuscular fat area (FAT, cm^2^). The fCSA and FAT were calculated by the Otsu thresholding technique in ImageJ, based on differences in the signal intensity. Pixels above the threshold were considered as fat, whereas pixels below the threshold were considered as lean muscle. The measurement technique has been described previously (Niemeläinen et al., 2011). Subsequently, the FI (%) was calculated applying the formula: $\text{FI}=\left(\frac{\text{FAT}}{\text{CSA}}\right)*$ 100. The right and left sides of the defined muscles were summarized and further normalized by the patients’ height (cm^2^/m^2^) as seen in recently published literature (Muellner et al., 2022). The measurements were performed by an orthopedic resident.

### 2.3 Spondylolisthesis measurements

All included patients received preoperative standard anterior-posterior and lateral as well as extension and flexion lumbar radiographs. Based on the lateral radiographs (standard, flexion and extension), the determination of the degree of spondylolisthesis was made according to the Meyerding classification (Meyerding, 1932). Furthermore, the extent of translation was determined from the radiographs using a line drawn on the posterior margin of the L5 vertebra. Using this line as a starting point, a vertical line was drawn at a 90° angle to the posterior inferior corner of the L4 vertebra and the translation was measured in millimeters (mm) as previously described by Shigematsu et al. (2010). All measurements were performed by an orthopedic resident.

### 2.4 Statistical analysis

Prior to further analysis, the continuous variables were tested for normal distribution by applying the Shapiro Wilk test. Due to a normal distribution of the variables, means and standard deviations (SD) are presented. Categorical variables are presented as total numbers (=N). To compare continuous variables within two groups, the *t*-test was applied. Categorical variables were compared using the Fisher's exact test. Pearson correlation coefficient was determined to assess the relationship between muscular parameters and the translation of the L4 vertebra. A multiple linear regression model was performed, with the translation of the L4 vertebra as the dependent variable. The independent variables of the model were age, sex, BMI, FI_MU_, FI_ES_ and FI_PS_. The model was tested for multicollinearity (variance influence factor = VIF) and independency of residuals using the Durbin-Watson statistic. We considered a VIF below 5 and a Durbin Watson statistic between 1.5 and 2.5 as acceptable. The residuals were tested for normal distribution and homoscedasticity. Statistical significance was defined as a p-value < 0.05. All analyses were conducted using SPSS Version 28.0 (IBM Corporation, Armonk, NY, United States).

## 3 Results

### 3.1 Patient demographics

**Fig. 2:**
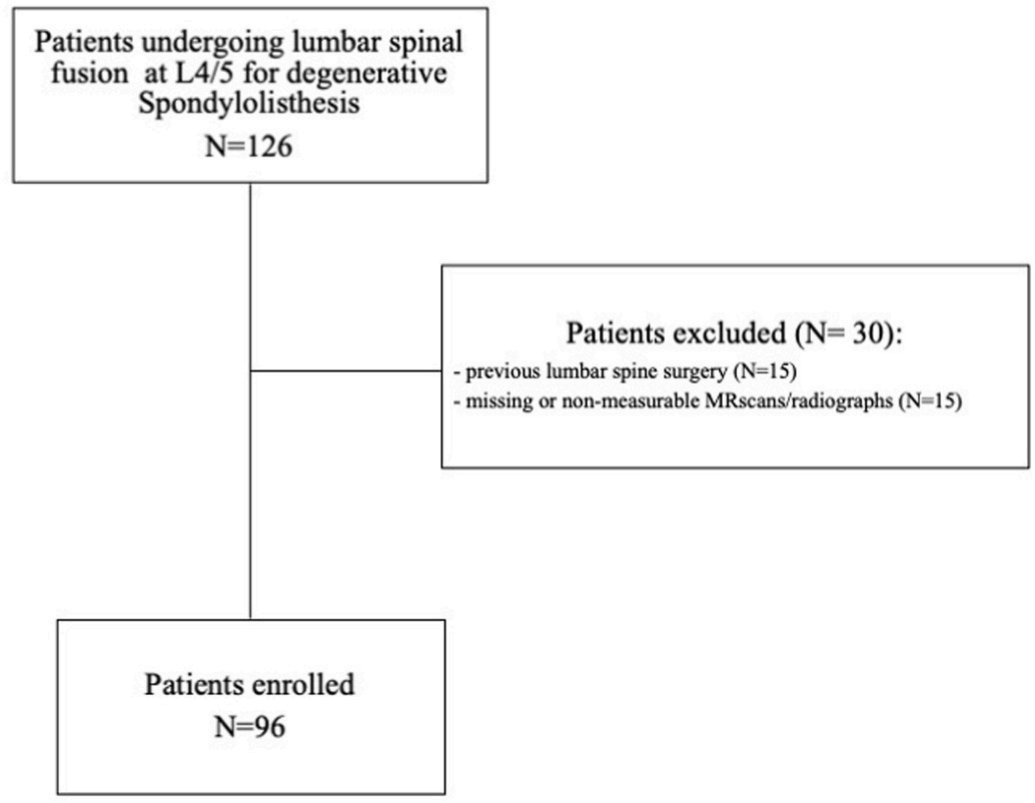
Flow chart of patient inclusion.

A total of 126 patients who underwent lumbar spinal fusion of only the L4/5 level for DLS were identified. Of these, 30 were excluded due to previous spine surgery and missing or non-measurable image data, yielding 96 enrolled patients (Fig. 2), 61 (62.5%) of which were female.

Patient demographics according to patient sex are summarized in Table 1. Female patients showed a significantly higher frequency of spondylolisthesis Meyerding grades > 1 (p = 0.018) while their mean L4 translation was larger than in male patients but not significantly different (7.53 ± 2.89 vs. 6.5 ± 2.91 mm, p = 0.099).

**Table 1:** Patient demographics for the total cohort and for female and male patients. p-values are given for comparisons between female and male patients and significant values are marked in bold. BMI, body mass index; COPD, chronic obstructive pulmonary disease; ASA, American Society of Anesthesiology score; rec, reclination; inc, inclination.

	All	Female	Male	p-value (f/m)
N	96	61	35	-
Age [years]	69.72 (±10.2)	68.7 (±10.02)	71.49 (±10.39)	0.205
BMI [kg/m^2^]	27.69 (±4.85)	27.4 (±4.97)	28.21 (±4.67)	0.209
Height [cm]	168.17 (±8.42)	163.8 (±6.3)	175.8 (±6.3)	**<0.001**
Weight [kg]	78.54 (±15.84)	73.3 (±12.4)	87.54 (±17.17)	**<0.001**
**Comorbidities**				

Diabetes	14/96	8/61	6/35	0.399
Hypertension	64/96	37/61	27/35	0.076
COPD	4/96	0/61	4/35	**0.015**
Congestive heart failure	9/96	3/61	4/35	0.217
**ASA**	85	54	31	**-**

I	5/85	4/54	1/31	0.733
II	48/85	30/54	18/31	
III	32/85	20/54	12/31	
**Meyerding**				

1	72	40	32	**0.018**
2	23	20	3	
3	1	1	0	
Translation [mm]	7.16 (±2.92)	7.53 (±2.89)	6.5 (±2.91)	0.099
Transl. rec [mm]	7.37 (±3.34)	7.91 (±3.56)	6.45 (±2.85)	**0.042**
Trans. inc [mm]	9.32 (±3.76)	9.55 (±3.9)	8.93 (±3.89)	0.464

### 3.2 Muscle degeneration according to patient sex

There were no significant differences in the multifidus or erector spinae muscles’ CSA, fCSA, or FI between female and male patients (Table 2). In the psoas muscle, female patients showed a significantly lower CSA (9.61 ± 3.18 vs. 12.32 ± 3.47, p < 0.001) and fCSA (9.50 ± 3.12 vs. 12.12 ± 3.44, p < 0001) while there were no significant differences in FI.

**Table 2:** Muscle measurements for the paraspinal muscles. p-values are given for comparisons between female and male patients and significant values are marked in bold. CSA, cross-sectional area; fCSA, functional cross-sectional area; FAT, intramuscular fat area; FI, fatty infiltration.

		All N = 96	Female N = 61	Male N = 35	p-value (f/m)
Multifidus	CSA [cm^2^/m^2^]	8.88 (±2.29)	8.88 (±2.37)	8.91 (±2.16)	0.947
	fCSA [cm^2^/m^2^]	5.94 (±2.14)	5.80 (±2.24)	6.19 (±1.96)	0.387
	FAT [cm^2^/m^2^]	2.94 (±1.53)	3.08 (±1.65)	2.71 (±1.29)	0.267
	FI [%]	33.40 (±15.16)	35.03 (±15.87)	30.57 (±13.60)	0.168
Erector Spinae	CSA [cm^2^/m^2^]	13.71 (±4.10)	14.27 (±4.36)	12.74 (±3.46)	0.077
	fCSA [cm^2^/m^2^]	11.46 (±3.85)	11.82 (±4.02)	10.84 (±3.50)	0.228
	FAT [cm^2^/m^2^]	2.25 (±1.59)	2.45 (±1.70)	1.90 (±1.32)	0.102
	FI [%]	16.98 (±12.49)	17.75 (±12.89)	15.63 (±11.82)	0.425
Psoas	CSA [cm^2^/m^2^]	10.60 (±3.52)	9.61 (±3.18)	12.32 (±3.47)	**<0.001**
	fCSA [cm^2^/m^2^]	10.45 (±3.47)	9.50 (±3.12)	12.12 (±3.44)	**<0.001**
	FAT [cm^2^/m^2^]	0.145(±0.33)	0.11 (±0.29)	0.20 (±0.39)	0.237
	FI [%]	1.32 (±2.49)	1.17 (±2.95)	1.59 (±2.81)	0.433

### 3.3 Association between muscle degeneration and L4 translation

In the correlation analysis, the FI of neither the multifidus, the erector spinae nor the psoas muscles showed a significant correlation with the extent of L4 translation (Table 3).

**Table 3:** Correlation analysis for the correlation between the fatty infiltration (FI) of the multifidus, erector spinae, or psoas muscles and the extent of L4 translation.

		FI_MU_ [%]	FI_ES_ [%]	FI_PS_ [%]
Translation [mm]	R	0.119	−0.038	−0.146
	p	0.248	0.710	0.155

The multiple linear regression model showed a significant positive association between the FI of the multifidus muscles with the extent of L4 translation while no significant influence was shown for any of the other variables included in the model (Table 4).

**Table 4:** Multiple linear regression analysis with the extent of L4 translation as the dependent variable. Significant p-values are marked in bold. The model has acceptable auto-correlation with a Durbin-Watson statistic of 1.841.

Predictor	*b* [*95%-CI*]	*SE*	Beta	*t*	*p*	VIF
(Constant)	8.523 [4.216; 12.830]	2.168		3.931	<0.001	
Age [years]	−0.045 [-0.110; 0.020]	0.032	−0.157	−1.386	0.169	1.315
Sex	0.604 [-0.629; 1.836]	0.620	0.100	0.973	0.333	1.080
FI_MU_ [%]	0.084 [0.022; 0.147]	0.032	0.436	2.668	**0.009**	2.743
FI_ES_ [%]	−0.070 [-0.143; 0.003]	0.037	−0.298	−1.899	0.061	2.528
FI_PS_ [%]	−0.180 [-0.419; 0.059]	0.120	−0.153	−1.493	0.139	1.075

Note. R^2^ = 0.123. (p = 0.034) Durbin-Watson Statistics: 1.841.

## 4 Discussion

Our study investigated the role of paraspinal muscle composition on the extent of translation in DLS of the L4/5 segment. We found a significant influence of the multifidus but not the erector spinae or psoas muscles’ FI on L4 translation. Thus, muscle composition may play a role in the pathogenesis of DLS. Interestingly, despite the more severe spondylolistheses found in female patients, neither patient sex nor patient age had a significant influence on L4 translation.

Our findings regarding the influence of the FI of the multifidus muscle on L4 translation are supported by a previous finite element study by Zhu et al. which shows that a reduction of muscular force is associated with the development or aggravation of DLS (Zhu et al., 2017). In a clinical analysis, Li et al. showed that both BMI and the degree of FI of the multifidus were independent risk factors for the development of DLS but did not investigate their association with the extent of vertebral slippage (Li et al., 2022). Furthermore, previous studies have shown an association between DLS and segmental muscle atrophy of the multifidus muscle (Kalichman et al., 2017; Wang et al., 2015). Wang et al. therefore conclude that multifidus atrophy and the associated reduced muscle strength may result in segmental instability and thereby spondylolisthesis. At the same time, they discuss that multifidus atrophy may be a result of spondylolisthesis. They also found a hypertrophy of the erector spinae muscle which they assume to be a compensatory mechanism to replace the multifidus muscle's function (Wang et al., 2015). Interestingly, while there seems to be a negative correlation between paraspinal muscle density and BMI, this is not found in the last two lumbar levels, which may indicate that muscle changes are induced by the pathology or the associated pain rather than the FI causing the pathology (Kalichman et al., 2017; Kjaer et al., 2007; Parkkola and Kormano, 1992). Both an increase in multifidus atrophy and a hypertrophy of the erector spinae muscle have been reported in isthmic spondylolisthesis as well (Thakar et al., 2016).

The multifidus muscle plays an important role in maintaining spinal stability and has been shown to have a higher percentage of Type I muscle fibers compared to other paraspinal muscles (Spratt et al., 1993). These Type I fibers are more vulnerable to pain and immobilization (Appell, 1990). As the patients included in our study received surgery due to persistent symptoms related to spondylolisthesis despite conservative treatment, the multifidus’ increased FI may be associated with chronic low back pain. Our analysis does not allow any conclusive statement whether this increase in multifidus FI is the cause or effect of a larger L4 translation but it seems that patients suffering from DLS may benefit from targeted strengthening of the multifidus muscle. While it has been shown, that specific motor re-education exercises cause a normalization of multifidus areas in patients with acute back pain (Hides et al., 1994), the impact of targeted multifidus strengthening has yet to be shown in patients with DLS. According to our results it seems possible that it may play a role in its radiological progression.

Multiple studies have shown the association between muscle health and surgical outcome after spine surgery (Han et al., 2023; Eksi et al., 2025; Chen et al., 2025). A recent meta-analysis found a higher FI of the multifidus to be correlated with poorer Oswestry Disability Index (ODI) scores and persistent low back pain after lumbar spine surgery (Han et al., 2023). Thus, they conclude that FI may be used for clinical decision-making as it may predict postoperative outcomes. Regarding DLS, one study was included in the meta-analysis showing that both the multifidus’ CSA ratio and FI were associated with ODI scores and low back pain after lateral lumbar interbody fusion (Chen et al., 2022). As our results also indicate that increased FI of the multifidus is associated with a higher degree of slippage, muscle morphology needs to be taken into consideration to adequately treat DLS. This is not only important for conservative treatment but, as recently prehabilitative measures including physiotherapy have increasingly been incorporated into the preoperative preparation of patients undergoing spine surgery, also needs to be incorporated into these concepts.

In line with the existing literature, our study consisted of more female than male patients (Wang et al., 2017). In our multiple regression model, however, sex showed no significant influence on the extent of L4 translation. Furthermore, as previously shown by Thakar et al., we also found a reduced CSA of the psoas muscle in female patients but no significant differences in the CSA of the multifidus or erector spinae muscles (Thakar et al., 2016).

Consistent with recent evidence, our findings highlight the clinical relevance of multifidus fatty infiltration in the context of segmental instability. The observed association between the degree of fatty infiltration and the extent of vertebral slippage carries important implications for both prehabilitation and postoperative rehabilitation strategies. Our results suggest that patients presenting with higher grades of spondylolisthesis and pronounced multifidus degeneration may benefit from targeted preoperative muscle-conditioning programs. These could include specific core-stabilization exercises, neuromuscular training, and individualized protocols aimed at improving lumbar extensor function. Implementing such prehabilitation strategies may enhance postoperative functional outcomes, shorten recovery time, and reduce the risk of persistent low back pain. Future studies should therefore explore whether structured, imaging-informed training interventions—particularly those guided by multifidus degeneration metrics—can be systematically integrated into clinical decision-making.

The observed association between multifidus fatty infiltration and vertebral translation raises the question of directionality—a relationship that is likely bidirectional and mutually reinforcing. Fatty infiltration progressively compromises the multifidus' capacity to generate intersegmental stiffness and dynamic stabilization, potentially facilitating listhesis progression over time. Conversely, chronic pain associated with spondylolisthesis may lead to voluntary or reflex-mediated unloading of the lumbar spine, promoting disuse atrophy and secondary fatty infiltration independent of primary degenerative change. These two mechanisms are not mutually exclusive; rather, they may interact in a self-perpetuating cycle in which muscle degeneration and segmental instability amplify one another. This interplay underscores the clinical relevance of paraspinal muscle quality as a potential therapeutic target, as interventions aimed at preserving or restoring multifidus function—such as targeted physiotherapy or minimally invasive stabilization—may interrupt this cycle and favorably influence disease trajectory.

Our study has several limitations. First, the retrospective study design is inherently associated with a potential selection bias. Second, we only included patients who were operated on and are therefore not able to make any statement on conservatively treated DLS. Furthermore, this analysis is limited to radiological parameters as due to the retrospective design, we were not able to include validated clinical outcome measures. A key limitation of this study is the inclusion of only surgically treated DLS patients, which may introduce selection bias and limits generalizability to conservatively managed cohorts, particularly given the potentially different natural history of multifidus degeneration in earlier stages of degenerative spondylolisthesis. It should be acknowledged that the relatively low explanatory power of our regression model (R^2^ = 0.123) likely reflects the multifactorial etiology of paraspinal muscle degeneration and vertebral translation; additional confounders not captured in our dataset—including duration of symptoms, sagittal spinopelvic alignment parameters, and systemic comorbidities such as diabetes mellitus and osteoporosis—may further contribute to the observed variability and warrant investigation in future studies.

In conclusion, our results show an association between the FI of the multifidus muscle with the degree of L4 translation in DLS. While due to the retrospective observational character of our study we cannot provide a conclusive statement on whether the muscle degeneration is cause or effect of spinal instability, our findings support recent studies postulating that muscle morphology needs to be taken into consideration when treating degenerative lumbar pathologies. Future studies need to focus on the effect specific multifidus strengthening has on the outcome of both conservatively and surgically treated DLS. Beyond their scientific implications, the present findings carry direct clinical relevance for the preoperative management of degenerative lumbar spondylolisthesis. Quantitative assessment of paraspinal muscle quality—readily obtainable from routinely acquired MRI sequences without additional cost or radiation exposure—may serve as a valuable adjunct in preoperative planning and patient counseling. Patients exhibiting advanced multifidus fatty infiltration may represent a subgroup at higher risk for segmental instability and potentially inferior surgical outcomes, warranting individualized treatment strategies and more intensive postoperative rehabilitation protocols. Integrating muscle quality parameters into established preoperative scoring systems could further refine patient stratification and shared decision-making.

## Ethics approval

This study was performed in line with the principles of the Declaration of Helsinki. Approval was granted by the Ethics Committee of Charité – Universitätsmedizin Berlin (EA2/194/22).

## Consent

Informed consent and consent to participate were waived by the Ethics Committee due to the study's retrospective design.

## Data availability

The datasets generated and/or analyzed during the current study are not publicly available due to patient privacy but are available from the corresponding author on reasonable request.

## Authors’ contribution

Conceptualization: Maximilian Muellner, Friederike Schö; mig, Matthias Pumberger, Hendrik Schmidt, Alexander P. Hughes. Methodology: Maximilian Muellner, Friederike Schö; mig, Matthias Pumberger, Lukas Schönnagel, Ulrike Kielburg. Data curation: Maximilian Muellner, Friederike Schö; mig, Ulrike Kielburg. Formal analysis and investigation: Maximilian Muellner, Friederike Schö; mig, Lukas Schönnagel, Alexander P. Hughes, Matthias Pumberger. Writing - original draft preparation: Friederike Schö; mig. Writing - review and editing: Friederike Schö; mig, Maximilian Muellner, Ulrike Kielburg, Hendrik Schmidt, Alexander P. Hughes, Matthias Pumberger. Resources: Hendrik Schmidt, Matthias Pumberger. Supervision: Matthias Pumberger, Hendrik Schmidt, Friederike Schö; mig, Alexander P. Hughes.

## Funding

This study was funded by the German Research Foundation [SCHM 2572/11-1, SCHM 2572/13-1, PU 762/1-1].

## Conflicts of interests

The authors have no relevant financial or non-financial interests to disclose.
